# 

**Published:** 1902-12-06

**Authors:** 


					ise hospital. "The standard of highest purity."?Lcmcet. dec. gth, 1902.
CADBURY'S If* CADBURY'S
cocoa iFm M oocoa
ABSOLUTELY PURE. ^ NO DBUOS O* AUCAUBa USB*.
fro. 8451 Vol. XXXIII. [? NEwipaper!]
Saturday, Dec. 6th, 1902.
L Beep 15S. J PHIOH TWOPENCB.

				

